# Modeling of the Return Current in a Light-Addressable Potentiometric Sensor

**DOI:** 10.3390/s19204566

**Published:** 2019-10-21

**Authors:** Tatsuo Yoshinobu, Daisuke Sato, Yuanyuan Guo, Carl Frederik Werner, Ko-ichiro Miyamoto

**Affiliations:** 1 Department of Biomedical Engineering, Tohoku University, 6-6, Aza-Aoba, Aramaki, Aoba-ku, Sendai 980-8579, Japan; 2 Department of Electronic Engineering, Tohoku University, 6-6, Aza-Aoba, Aramaki, Aoba-ku, Sendai 980-8579, Japan; 3 Frontier Research Institute of Interdisciplinary Sciences, Tohoku University, 6-3 Aza-Aoba, Aramaki, Aoba-ku, Sendai 980-8578, Japan

**Keywords:** light-addressable potentiometric sensor, LAPS, chemical imaging sensor, field-effect sensor

## Abstract

A light-addressable potentiometric sensor (LAPS) is a chemical sensor with a field-effect structure based on semiconductor. Its response to the analyte concentration is read out in the form of a photocurrent generated by illuminating the semiconductor with a modulated light beam. As stated in its name, a LAPS is capable of spatially resolved measurement using a scanning light beam. Recently, it has been pointed out that a part of the signal current is lost by the return current due to capacitive coupling between the solution and the semiconductor, which may seriously affect the sensor performance such as the signal-to-noise ratio, the spatial resolution, and the sensitivity. In this study, a circuit model for the return current is proposed to study its dependence on various parameters such as the diameter of contact area, the modulation frequency, the specific conductivity of the solution, and the series resistance of the circuit. It is suggested that minimization of the series resistance of the circuit is of utmost importance in order to avoid the influence of the return current. The results of calculation based on this model are compared with experimental results, and its applicability and limitation are discussed.

## 1. Introduction

**Figure 1:**
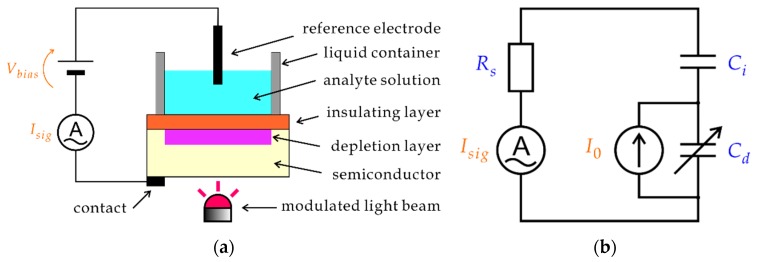
(**a**) Schematic of a LAPS. A bias voltage is applied to the field-effect structure so that a depletion layer is formed. The thickness of the depletion layer varies with the analyte concentration. The semiconductor substrate is illuminated with a modulated light beam, and the ac photocurrent signal $I_{sig}$ is measured and correlated to the analyte concentration. (**b**) A circuit model of a LAPS. The internal current $I_{0}$ is divided by the capacitance of the depletion layer $C_{d}$ and that of the insulating layer $C_{i}$ connected to the input resistance of the circuit $R_{s}$.

A light-addressable potentiometric sensor (LAPS) [1,2,3] is a semiconductor-based chemical sensor, which has a field-effect structure shown in Figure 1a. A dc voltage is applied to induce a depletion layer, the thickness of which varies due to the field effect in response to the analyte concentration on the sensing surface. A photocurrent generated by illuminating the semiconductor is measured to detect the variation of the capacitance of the depletion layer and to determine the analyte concentration. A spatially resolved measurement is possible by using a scanning light beam, which is the basis of the chemical imaging sensor [3,4] and the scanning photo-induced impedance microscopy [5].

In theoretical analysis of a LAPS, a simple circuit model, shown in Figure 1b, has been conventionally employed [6,7,8]. In this model, separation of electrons and holes by the electric field inside the depletion layer is represented by an internal ac current source $I_{0}$, which is divided by the capacitance of the depletion layer $C_{d}$ and that of the insulating layer $C_{i}$ connected to the series resistance of the circuit $R_{s}$. Here, $R_{s}$ consists of the resistance of the solution between the illuminated point and the reference electrode, the resistance of the reference electrode (in case the counter electrode is not used), the input resistance of the ammeter, and the contact resistance on the back surface of the semiconductor substrate. When the thickness of the depletion layer changes in response to the analyte concentration of the solution in contact with the sensing surface, variation of $C_{d}$ results in variation of the signal current $I_{sig}$. This circuit model was further combined with the carrier diffusion model [8,9,10,11,12,13] to describe the operation of the chemical imaging sensor, taking account of lateral diffusion and recombination of minority carriers inside the semiconductor substrate.

**Figure 2:**
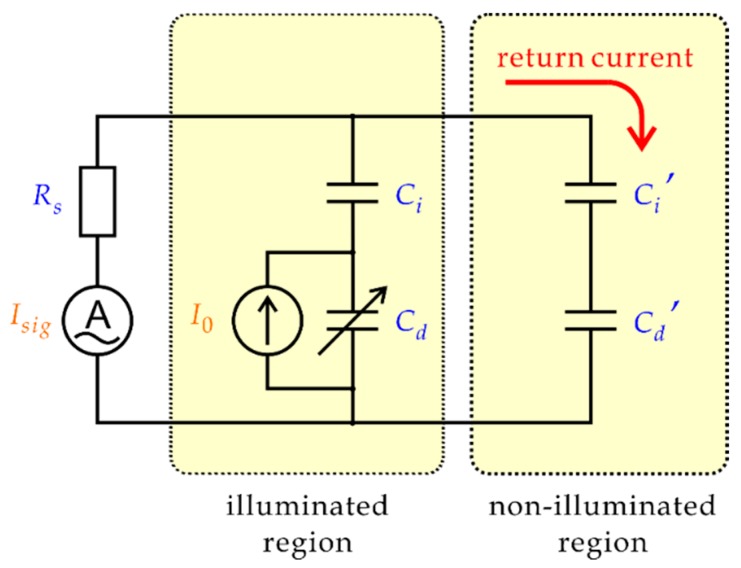
A simple circuit model of the return current. Due to the capacitive coupling of the solution and the semiconductor substrate, a part of the ac photocurrent returns to the semiconductor substrate through the capacitance of the insulating layer and that of the depletion layer in the non-illuminated region without contributing to the signal current $I_{sig}$.

Although these models were successful in describing important features of the chemical imaging sensor including its spatial resolution and frequency characteristics, Poghossian et al. [14] pointed out the influence of capacitive coupling between the solution and the semiconductor substrate in the non-illuminated region, which was not included in existing models. Figure 2 shows the simplest model, in which a part of the ac current returns to the semiconductor substrate through ${C_{i}}^{\prime }$ and ${C_{d}}^{\prime }$ without contributing to the signal current $I_{sig}$. It should be noted that this effect applies only to an ac current. In light-activated electrochemistry (LAE) [15], which has a similar setup to that of LAPS but uses dc faradaic current, the high impedance of the non-illuminated region separates the solution and the substrate. Figure 2 gives only an intuition that the capacitive coupling increases with the area of the non-illuminated region and the frequency of the ac current. It can be easily speculated that a loss of the signal current due to the return current may have a large impact on the signal-to-noise ratio, the spatial resolution, and the sensitivity. To be able to understand the dependence of the return current on various parameters and to evaluate its impact on the sensor performance, the model shown in Figure 2 is far too simple.

In this study, a new circuit model is proposed, in which the path of the return current is described as a transmission line. The model is used to calculate the influence of the return current on $I_{sig}$ and its dependence on parameters such as the frequency, the size of the non-illuminated region, the conductivity of the solution, and the series resistance of the circuit. Applicability and limitation of the model are discussed by comparing the results obtained by calculation and measurement.

## 2. Model

**Figure 3:**
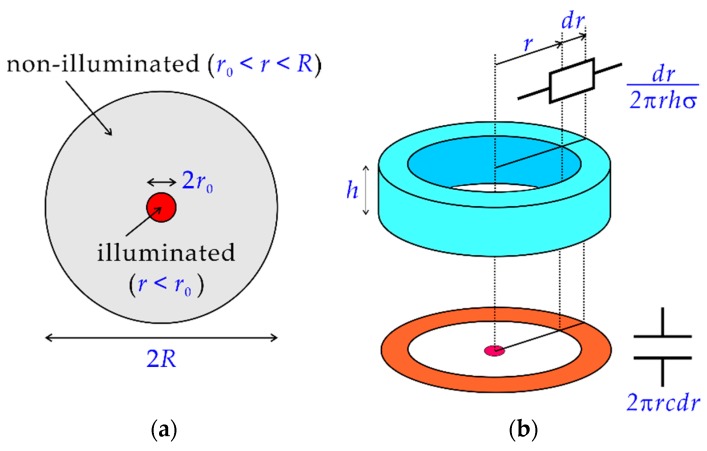
(**a**) Top view of the sensing surface in contact with the solution. A part of the photocurrent generated inside the illuminated region ($r<r_{0}$) returns to the semiconductor substrate through the non-illuminated region ($r_{0}<r<R$) by capacitive coupling. (**b**) The resistance of the solution between the inner and outer walls of a hollow cylinder and the combined capacitance of the insulating layer and the depletion layer under the sensing surface in a ring shape are considered.

Figure 3a shows the top view of the model, in which a circular region on the sensing surface with a radius R is in contact with the solution and a circular region with a radius $r_{0}$ at the center is illuminated. The rest of the contact area ($r_{0}<r<R$) is non-illuminated. The resistance of the solution in an infinitesimal volume between the inner and outer walls of a hollow cylinder with a radius r and a thickness dr shown in the upper part of Figure 3b is given by:

(1) $$\frac{dr}{2\pi rh\sigma },$$

where h is the height of the solution and σ is the specific conductivity of the solution. The combined capacitance of the insulating layer and the depletion layer in an infinitesimal area between r and r+dr shown in the lower part of Figure 3b is given by

(2) 2πrcdr,

where c is the combined capacitance per unit area. When the capacitance of the insulating layer per unit area $c_{i}$ and that of the depletion layer $c_{d}$ are connected in series, the combined capacitance is given by

(3) $$c=\frac{1}{\frac{1}{c_{i}}+\frac{1}{c_{d}}}.$$

**Figure 4:**
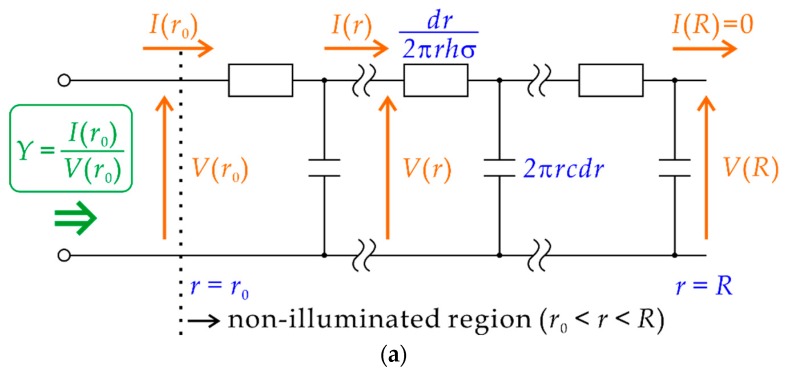

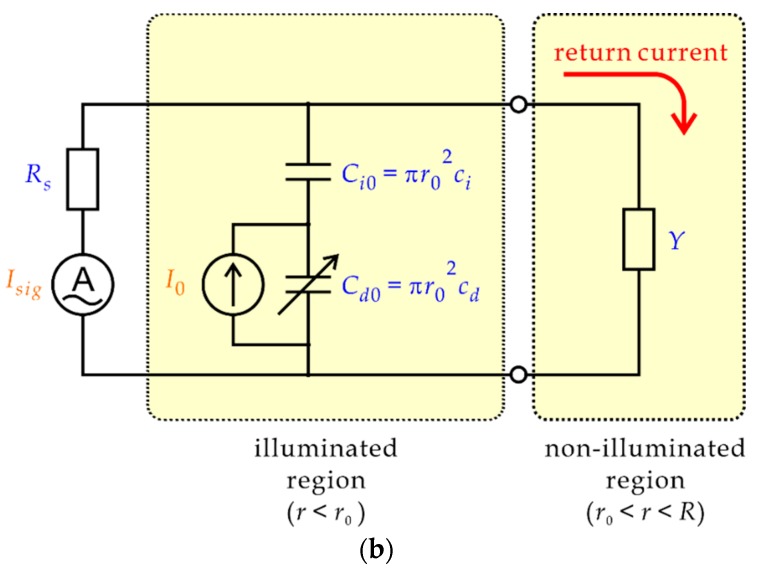
(**a**) A circuit model of the non-illuminated region. The resistance of the solution in an infinitesimal volume and the capacitance in an infinitesimal area shown in Figure 3b are connected in a ladder network in the range of $r_{0}<r<R$. By solving the telegraph equations of the transmission line under the initial conditions at r=R, the final values at $r=r_{0}$ are obtained, from which the input admittance Y is obtained. (**b**) A circuit model, in which the path of the return current is represented by admittance Y.

The admittance of the non-illuminated region Y is represented as a ladder network shown in Figure 4a, which is essentially a finite-length transmission line with an open end. Unlike a conventional transmission line, however, the resistance and the capacitance per unit length are not constant but dependent on r as described by (1) and (2), respectively. The telegraph equations of the transmission line are

(4a) $$I^{\prime }\left(r\right)=-j\omega 2\pi rcV\left(r\right),$$

(4b) $$\;V^{\prime }\left(r\right)=-\frac{1}{2\pi rh\sigma }I\left(r\right),\;$$

where I(r) and V(r) are complex numbers representing the phasors of the ac current and the ac voltage with an angular frequency ω (=2πf) at position r and j is the imaginary unit. From Equations (4a) and (4b) we obtain a second-order ordinary differential equation,

(5) $$I^{\prime \prime }\left(r\right)=\frac{1}{r}I^{\prime }\left(r\right)+jkI\left(r\right),$$

where

(6) $$k=\frac{\omega c}{h\sigma }.$$

By solving Equation (5) under initial conditions at r=R, we can obtain final values $I\left(r_{0}\right)$ and $I^{\prime }\left(r_{0}\right)$. Then, we also obtain $V\left(r_{0}\right)$ from Equation (4a), and the input admittance Y in Figure 4b is given by

(7) $$Y=\frac{I\left(r_{0}\right)}{V\left(r_{0}\right)}.$$

Once Y is obtained, the signal current $I_{sig}$ can be calculated as follows.

(8) $$I_{sig}=I_{0}\times \frac{1}{\left(1+\frac{C_{d0}}{C_{i0}}\right)\left(1+R_{s}Y\right)+j\omega C_{d0}R_{s}}.$$

Here, $C_{i0}$ is the capacitance of the insulating layer inside the illuminated region and $C_{d0}$ is that of the depletion layer, which are given by

(9) $$C_{i0}=\pi r_{0}{}^{2}c_{i},$$

(10) $$\;C_{d0}=\pi r_{0}{}^{2}c_{d}.\;$$

The initial conditions at r=R are given as follows. Since the current does not flow out of the contact area,

(11) I(R)=0.

The value of $I^{\prime }\left(R\right)$, or equivalently the value of V(R), can be arbitrarily given, as we are interested only in the ratio of I(r) and V(r). For simplicity, we choose it to be

(12) $$I^{\prime }\left(R\right)=-1.$$

For ease of calculation, the second-order ordinary differential equation of a complex-valued function (5) can be converted into a set of first-order ordinary differential equations of four real-valued functions by defining

(13a) y1(r)=Re I(r),

(13b)  y2(r)=Im I(r), 

(13c) $$\;y_{3}\left(r\right)={y_{1}}^{\prime }\left(r\right),\;$$

(13d) $$\;y_{4}\left(r\right)={y_{2}}^{\prime }\left(r\right).\;$$

Then, our problem is to solve a set of differential equations

(14a) $${y_{1}}^{\prime }\left(r\right)=y_{3}\left(r\right),$$

(14b) $$\;{y_{2}}^{\prime }\left(r\right)=y_{4}\left(r\right),\;$$

(14c) $$\;{y_{3}}^{\prime }\left(r\right)=\frac{1}{r}y_{3}\left(r\right)-ky_{2}\left(r\right),\;$$

(14d) $$\;{y_{4}}^{\prime }\left(r\right)=\frac{1}{r}y_{4}\left(r\right)+ky_{1}\left(r\right),\;$$

under the initial conditions

(15a) $$y_{1}\left(R\right)=0,$$

(15b) $$\;y_{2}\left(R\right)=0,\;$$

(15c) $$\;y_{3}\left(R\right)=-1,\;$$

(15d) $$\;y_{4}\left(R\right)=0.\;$$

Finally, we obtain the values $y_{1}\left(r_{0}\right)$, $y_{2}\left(r_{0}\right)$, $y_{3}\left(r_{0}\right)$, and $y_{4}\left(r_{0}\right)$, which give

(16a) $$I\left(r_{0}\right)=y_{1}\left(r_{0}\right)+jy_{2}\left(r_{0}\right),$$

(16b) $$\;V\left(r_{0}\right)=\frac{1}{\omega 2\pi r_{0}c}\left\{-y_{4}\left(r_{0}\right)+jy_{3}\left(r_{0}\right)\right\}.\;$$

In the following sections, calculations were done by a Runge–Kutta solver *ode45* of MATLAB^®^ (MathWorks). Parameters listed in Table 1 were used so that the results of calculation can be compared with those experimentally obtained.

## 3. Dependence on R and f 

**Figure 5:**
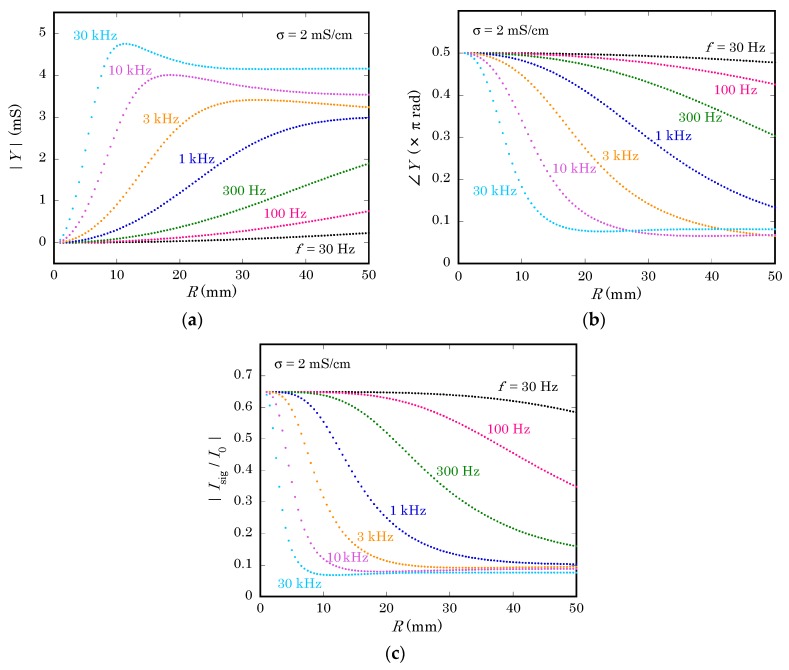
Dependence of (**a**) the magnitude and (**b**) the argument of the input admittance of the non-illuminated region Y, and (**c**) the ratio of the signal current $I_{sig}$ to the internal current $I_{0}$ on the radius of the non-illuminated region R at different modulation frequencies of the light beam f.

First of all, the dependence of the admittance Y on the radius of the non-illuminated region R and the frequency f was calculated as summarized in Figure 5.

Figure 5a shows the magnitude of Y calculated with the model described in the previous section. As expected, the magnitude of Y becomes larger at higher frequencies, meaning that more current returns from the solution to the semiconductor substrate through their capacitive coupling. Moreover, the magnitude of Y becomes larger as R increases and the contact area becomes larger. However, the admittance of a finite-length transmission line does not always increase monotonously with its length. In fact, curves for the frequency of 3 kHz and higher have maxima.

In Figure 5b, the argument of Y is plotted as a function of R. When the contact area is small, the transmission line is mostly capacitive, and it becomes more resistive as R increases. This behavior is qualitatively understood as follows. When R increases, more portion of the return current flows through the capacitance at locations further from the center, in other words, after going through a larger lateral resistance of the solution on the way. At higher frequencies, this transition of the argument of Y occurs at smaller distance R.

Figure 5c shows the magnitude of $I_{sig}/I_{0}$ as a function of R at different frequencies. As R increases, the admittance of the non-illuminated region Y becomes larger and the signal current decreases due to the increase of the return current. In the present case, where calculation was done with parameters listed in Table 1, the signal current decreased down to about 14% of the value at $R=r_{0}$, where the non-illuminated region does not exist.

## 4. Dependence on σ and $R_{s}\;$

**Figure 6:**
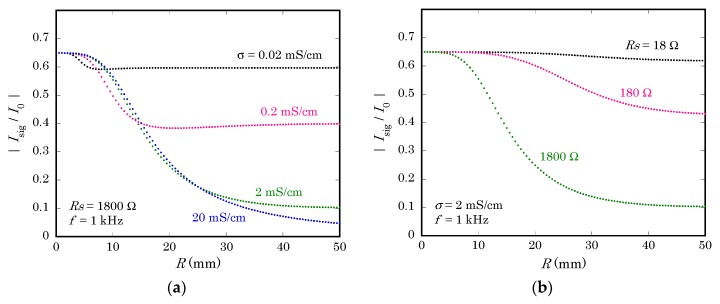
The magnitude of $I_{sig}$ calculated for different values of (**a**) the specific conductivity of the solution σ and (**b**) the resistance of the circuit $R_{s}$.

In Figure 6a, the dependence of the signal current $I_{sig}$ on the radius of the non-illuminated region R is plotted for different values of the specific conductivity of the solution σ. Here, the series resistance of the circuit $R_{s}$ and the frequency f were kept constant at 1800 Ω and 1 kHz, respectively. When the solution is less conductive, the effect of the return current is relatively smaller, because the lateral resistance will limit the distance from the center, within which the capacitive coupling contributes to the return current. It should be noted, however, that a smaller conductivity of the solution also implies a higher resistance of the solution between the illuminated point and the reference electrode, which increases $R_{s}$. As a whole, therefore, less conductivity will not necessarily reduce the effect of the return current.

In Figure 6b, calculation was done for different values of $R_{s}$, while keeping σ and f at 2 mS/cm and 1 kHz, respectively. It clearly shows that reduction of $R_{s}$ is of utmost importance in removing the effect of the return current. To reduce $R_{s}$, a three-electrode system with a counter electrode should be used to bypass the resistance of the reference electrode. The electronic circuit to collect the ac photocurrent signal, typically a transimpedance amplifier based on operational amplifiers, should be carefully designed to minimize its input impedance. The ohmic contact on the back surface of the semiconductor substrate must be carefully formed to minimize the contact resistance.

In some applications, a planer counter electrode can be placed in parallel to the sensing surface, so that the vertical distance from the illuminated point to the counter electrode is always small and constant even when the position of the light beam is moved for scanning. In case of measurement inside a microfluidic device, a metallic wire can be inserted along the microchannel as a counter electrode. In these cases, the counter electrode helps, on one hand, to reduce $R_{s}$, but it also shortcuts the lateral resistance of the solution and delivers the return current to locations far from the illuminated point and may increase the return current.

## 5. Impact on the Sensitivity

In chemical imaging based on a LAPS, a focused light beam scans the semiconductor substrate, and the signal current is recorded at each pixel. The signal current is then converted into the analyte concentration using a calibration curve acquired prior to the measurement. For a small change, a linear approximation is used to convert a variation of the signal current into that of the potential, which is then linearly correlated to the logarithm of the activity of the analyte using the Nernst equation.

Under the existence of a return current, however, this conversion may be systematically affected due to the following reason. During the calibration step, the entire sensing surface is uniformly in contact with known concentrations of analyte solutions. In such a case, the thickness of the depletion layer varies equally both in the illuminated region and in the non-illuminated region, and $I_{sig}$ varies under the global change of $c_{d}$. During the measurement step, however, $c_{d}$ may change only locally, and the return current may be different from that in the calibration step.

**Figure 7:**
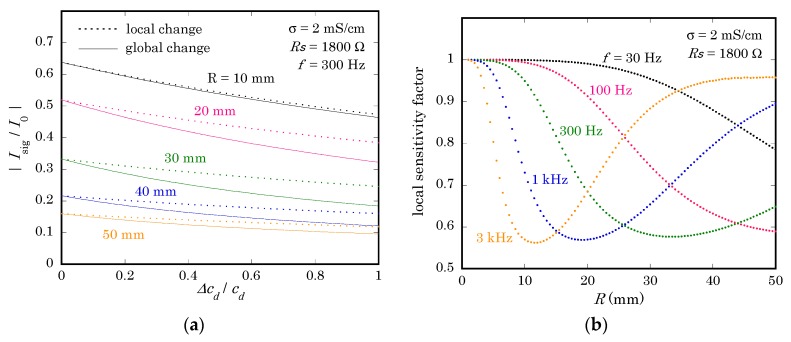
(**a**) Variation of $I_{sig}$ as a function of $\Delta c_{d}/c_{d}$ for a global change of $c_{d}$ (solid lines) and for a local change of $c_{d}$ (dotted lines). The values of σ, $R_{s}$, and f were 2 mS/cm, 1800 Ω, and 300 Hz, respectively. (**b**) The local sensitivity factor calculated for different values of R and f. The values of σ and $R_{s}$ were 2 mS/cm and 1800 Ω, respectively.

To illuminate the difference, calculation was carried out in two different situations. First, $I_{sig}$ was calculated while changing $c_{d}$ both in the illuminated region and in the non-illuminated region, which corresponds to the situation of the calibration step. Second, $I_{sig}$ was calculated while changing $c_{d}$ only in the illuminated region with $c_{d}$ in the non-illuminated region unchanged. Figure 7a shows, for different values of R, the variation of $I_{sig}$ as a function of $\Delta c_{d}/c_{d}$ in the range of 0 to 1. It is clearly observed that the variation of $I_{sig}$ for a local change of $c_{d}$ is smaller than that for a global change of $c_{d}$. In other words, a local change of the analyte concentration in imaging will be underestimated due to the difference of the return current during calibration and measurement.

The ratio of the slope (calculated in the range between $\Delta c_{d}/c_{d}$ = 0 and 1) for a local change of $c_{d}$ in Figure 7a and that for a global change of $c_{d}$ was defined as a local sensitivity factor, which indicates the degree of underestimation. Figure 7b shows the local sensitivity factor as a function of R at different frequencies. This result shows that a local change can be underestimated, depending on the combination of R and f, by a factor even smaller than 0.6 in the calculated case, where $R_{s}$ was 1800 Ω. When $R_{s}$ was reduced to 18 Ω, the local sensitivity factor calculated within the same range of conditions as in Figure 7b was always larger than 0.96 (data not shown), meaning that the underestimation was less than 4%. This result again shows the importance of reducing $R_{s}$ in removing the effect of the return current in a LAPS.

## 6. Comparison with Experiments

A series of experiments were carried out to observe the dependence of $I_{sig}$ on R and f in a real situation. A large-area LAPS plate was prepared by depositing 50 nm SiO_2_ and 50 nm Si_3_N_4_ successively on the entire surface of a 6-inch n-type Si wafer with a thickness of 200 μm and a resistivity of 1–10 Ωcm. An ohmic contact was evaporated on the perimeter of the back surface. To define different sizes of contact areas between the solution and the sensing surface, various sizes of cylindrical liquid containers to accommodate the solution were prepared by a 3D printer and attached to the sensing surface via O-rings with inner diameters 21.0, 40.5, 60.5, 82.5, and 102.5 mm. The solution used in this experiment was 0.1 wt% NaCl solution with a specific conductivity σ = 2.0 mS/cm and the height was h = 10 mm. A Ag/AgCl reference electrode (RE-1B, BAS Inc.) was dipped into the solution at the center.

The capacitance of the insulating layer per unit area $c_{i}$ and the series resistance of the circuit $R_{s}$ were determined by the following method. A coil with an inductance 100 mH was inserted in the circuit and the sensor was biased at $V_{bias}$ = +1.0 V, where the depletion layer disappears. For each size of the contact area, the ac current in response to the application of a small ac voltage was recorded to find the resonance peak while scanning the frequency. The capacitance was calculated from the peak position, and the series resistance was calculated from the peak height. Then, the value of $c_{i}$ was determined to be $4.49\times 10^{-4}$ F/m^2^ by linear regression of the capacitance versus the contact area, and the average value of $R_{s}$ was $1.8\times 10^{3}$ Ω. The measured value of $c_{i}$ was close to $4.54\times 10^{-4}$ F/m^2^, a theoretical value for a double layer comprising 50 nm SiO_2_ and 50 nm Si_3_N_4_. The capacitance of the depletion layer per unit area $c_{d}$ at $V_{bias}$ = −2.0 V was determined to be $2.42\times 10^{-4}$ F/m^2^ by linear regression of the measured capacitance versus the contact area. The measured value of $c_{d}$ was close to $2.28\times 10^{-4}$ F/m^2^, a theoretical value for n-type Si with a donor concentration $N_{D}=4\times 10^{15}$ cm^−3^ under strong inversion at 300 K.

**Figure 8:**
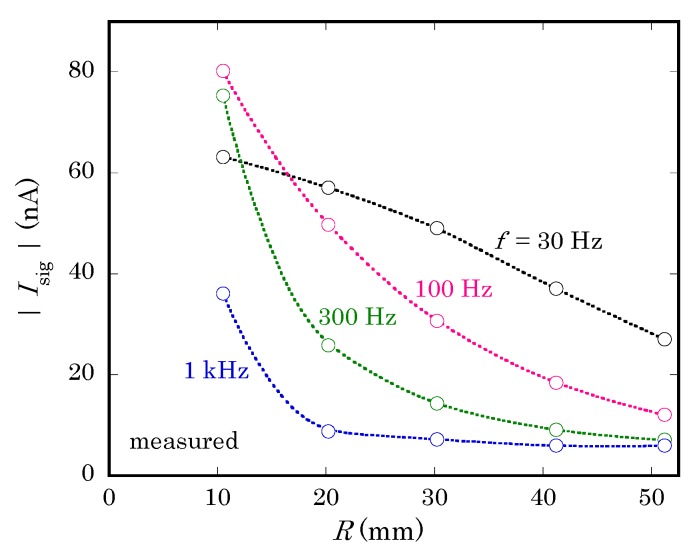
Experimentally obtained values of the magnitude of the ac current signal $I_{sig}$ at different values of the radius of the contact area R and the frequency f. The measurement was done in a 0.1 wt% NaCl solution with a specific conductivity of 2.0 mS/cm.

A LAPS signal was collected at $V_{bias}$ = −2.0 V by illuminating the center of the back surface with a modulated light beam from a red LED. The radius of illumination was restricted to 0.5 mm by an aperture. Figure 8 shows the magnitude of the ac current signal for different values of R and f. Here, it should be noted that the vertical axis of Figure 8 is not normalized, while the magnitude of $I_{sig}$ in Figure 5c is normalized to $I_{0}$, which depends on the frequency but cannot be directly measured in experiments. The similar dependence of curves on R and f in Figure 5c and Figure 8 proves that the loss of the current signal is qualitatively reproduced by calculating the return current using the proposed model.

A closer look at concavity of curves reveals that there is a discrepancy of frequencies by a factor of 2 to 3 between curves of corresponding shapes in Figure 5c and Figure 8. A possible reason of this discrepancy is the different waveforms of the ac photocurrent in calculation and experiments. While the calculation assumes an internal current source $I_{0}$ producing a sinusoidal waveform with a single frequency, the light beam used in an experiment is turned on and off at a certain frequency, which produces sinusoidal waveforms only at higher frequencies. When the frequency is relatively low, a transient current flows only for a short period after the light beam is turned on or off. The resulting waveform is distorted and contains higher frequency components [13], for which the susceptance becomes larger, and the return current will be larger than calculated.

The waveform of a LAPS current signal can be reproduced by a device simulation, which takes account of the dynamics of minority carriers inside the semiconductor layer [13]. For more precise estimation of the return current, therefore, combination of a circuit model and device simulation should be considered.

## 7. Conclusions

In this study, a circuit model for the return current in a LAPS was proposed, where the conductivity of the solution and the capacitive coupling between the solution and the semiconductor substrate in the non-illuminated region were formulated as a transmission line. The telegraph equation was numerically solved to find the input admittance of the non-illuminated region, and the dependence of the signal current on various parameters such as the diameter of contact area, the modulation frequency, the specific conductivity of the solution, and the series resistance of the circuit was investigated. It was found that a local change of the analyte concentration in imaging may be underestimated because of the difference of the return current in calibration and in measurement. The dependence of the LAPS signal current on the contact area and the frequency was also observed in experiments, which was compared with the calculated results.

## Figures and Tables

**Table 1:** Parameters used in calculation unless otherwise specified.

Parameter	Symbol	Value	Unit
Capacitance of the insulating layer per unit area	$\;c_{i}\;$	$\;4.49\times 10^{-4}\;$	$\;F/m^{2}\;$
Capacitance of the depletion layer per unit area	$\;c_{d}\;$	$\;2.42\times 10^{-4}\;$	$\;F/m^{2}\;$
Specific conductivity of the solution	σ	2	mS/cm
Series resistance of the circuit	$\;R_{s}\;$	1800	Ω
Radius of the illuminated region	$\;r_{0}\;$	0.5	mm
